# Supplementary material for: Sheep’s Butter and Correspondent Buttermilk Produced with Sweet Cream and Cream Fermented by Aromatic Starter, Kefir and Probiotic Culture
Source: Foods. 2023 Jan 10;12(2):331. doi: 10.3390/foods12020331 (PMC9857949; doi:10.3390/foods12020331)
Supplement: Supplementary file 1 [file foods-12-00331-s001.zip › foods-2053070-supplementary.pdf]

SUPPLEMENTARY MATERIAL

Table S1. Sensory characteristics of sheep's butters produced with sweet cream and with cream fermented with different starter cultures. Sensory scores of butters at the beginning (7 days) and at the end of storage (90 days).

Butter	Aroma		Taste		Texture		Rank	
	7 days	90 days	7 days	90 days	7 days	90 days	7 days	90 days
**S-Sweet**	3.94 ± 0.77	3.90 ± 1.12	4.03 ± 0.87	4.05 ± 0.94	4.06 ± 0.85	3.90 ± 1.07	2.23 ± 1.17	2.40 ± 1.27
**A-Aromatic**	3.58 ± 0.85	3.70 ± 1.03	3.77 ± 0.99	3.50 ± 1.19	3.87 ± 1.09	3.50 ± 1.19	2.57 ± 1.04	2.95 ± 1.0
**K-Kefir**	3.67 ± 0.76	3.95 ± 0.69	3.63 ± 1.07	3.85 ± 0.81	3.77 ± 0.94	3.95 ± 0.69	2.93 ± 1.17	2.30 ± 1.08
**P-Probiotics**	3.97 ± 0.75	3.75 ± 0.79	3.84 ± 0.86	3.85 ± 1.04	3.92 ± 0.73	3.60 ± 0.82	2.27 ± 1.01	2.35 ± 1.09

Table S2. Sensory characteristics of sheep's buttermilks produced with sweet cream and with cream fermented with different starter cultures.

Buttermilk	Aroma	Taste	Consistency	Rank	Global evaluation
**S-Sweet**	2.57 ± 1.04	2.67 ± 1.03	3.03 ± 1.03	2.67 ± 1.24	2.63 ± 0.93
**A-Aromatic**	2.83 ± 1.09	2.25 ± 1.01	2.67 ± 0.96	3.13 ± 0.94	2.35 ± 0.99
**K-Kefir**	3.27 ± 0.94	3.10 ± 0.92	3.77 ± 0.9	1.93 ± 0.98	3.30 ± 1.02
**P-Probiotics**	3.33 ± 1.12	2.60 ± 1.07	3.50 ± 0.82	2.23 ± 0.97	3.10 ± 0.92
